# Patient adherence to patient-reported outcome measure (PROM) completion in clinical care: current understanding and future recommendations

**DOI:** 10.1007/s11136-023-03505-y

**Published:** 2023-09-11

**Authors:** Elizabeth Unni, Theresa Coles, Danielle C. Lavallee, Jennifer Freel, Natasha Roberts, Kate Absolom

**Affiliations:** 1 Touro College of Pharmacy, New York, NY USA; 2 Duke University School of Medicine, Durham, NC USA; 3 Michael Smith Health Research BC, Vancouver, BC Canada; 4 University of Pittsburgh Medical Centre, Pittsburg, PA USA; 5 The University of Queensland Centre for Clinical Research, Herston, QLD Australia; 6 STARS Education and Research Alliance, Surgical Treatment and Rehabilitation Service (STARS), The University of Queensland and Metro North Health, Herston, QLD, Australia; 7 University of Leeds, Leeds, UK

**Keywords:** Patient-reported outcome measures (PROMs), Clinical practice, Adherence, Patient engagement

## Abstract

**Background:**

Patient-reported outcome measures (PROMs) are increasingly being used as an assessment and monitoring tool in clinical practice. However, patient adherence to PROMs completions are typically not well documented or explained in published studies and reports. Through a collaboration between the International Society for Quality-of-Life Research (ISOQOL) Patient Engagement and QOL in Clinical Practice Special Interest Groups (SIGs) case studies were collated as a platform to explore how adherence can be evaluated and understood. Case studies were drawn from across a range of clinically and methodologically diverse PROMs activities.

**Results:**

The case studies identified that the influences on PROMs adherence vary. Key drivers include PROMs administeration methods within a service and wider system, patient capacity to engage and clinician engagement with PROMs information. It was identified that it is important to  evaluate  PROMs integration and adherence from multiple perspectives.

**Conclusion:**

PROM completion rates are an important indicator of patient adherence. Future research prioritizing an understanding of PROMs completion rates by patients is needed.

## Plain English summary

This piece of work was done in response to a lack of literature on patient engagement with patient-reported outcome measures (PROMs) in clinical practice. At the International Society for Quality-of-Life 2019, a symposium session, and subsequent webinar, presented the available literature and findings from key case studies. It was identified is that there are both barriers and enablers that ultimately impact adherence by patients to completing PROMs. A focus on what motivates patients to complete these measures, and how engaged their clinicians are in the use of patient-reported outcomes in their clinical practice, was observed. Across the case studies, a variety of approaches were used to integrate PROMs into day-to-day care. This work has identified that the use of  such measures should be routinely evaluated by taking into consideration the experience of patients and clinicians, and the impacts on the clinical setting and health service. Key recommendations have been developed to reflect these findings.

## Background

Patient-Reported Outcomes Measures (PROMs) are collected by both researchers and clinical teams to understand patient experience of disease and treatment. Mounting evidence, in clinical research supports benefits for patients and health professionals when patient-reported data is used in clinical care, particularly to improve patient-clinician communication, patient satisfaction, and facilitation of meaningful focused conversations [1–5]. PROMs are increasingly used across a wide range of clinical settings for various reasons to inform patient care and maximize patient engagement in their own care. The use of these measures requires a series of crucial steps including: (i) deployment to the patient, (ii) collection/completion of the measure by the patient, (iii) tracking, (iv) review by clinician, (v) completion of these steps (e.g., an alert is closed out or results are discussed with a patient) [6]. Patient adherence to routine PROM completion is not fully understood. Technological advances (for example, interactive voice response/apps/web-based systems) continue to evolve, creating an increase in opportunities to streamline collection of patient-reported data, both within and outside the clinical care. For example, the Patient-Reported Outcome Measurement Information System (PROMIS) has increasing banks of measures, progressed computer adaptive testing (CAT) systems, using item response theory [7]. There are many factors influencing PROMs implementation [8].

Documented evidence regarding patient adherence to PROMs completion ranges from 50% [9–11] to above 80% [12, 13]. In a recent study done in 2022, it was observed that for longitudinal PROMs, the rate of completion significantly decreases with each additional timepoint captured. For example, following discharge, the completion rate of PROMs decreased to 68% (900 out of 1321 after 7 days, to 52% (671 out of 1288 after 3 months; and 25% (177 out of 709) after a year [14].

Individuals completing PROMs have better functional capacity on average [15]. Therefore, it is possible that missing data, may be not at random, instead indicates other factors including worsening health, inequitable access to healthcare and inequitable access to the internet for remote monitoring. For clinical teams, missing data can limit the ability of the PROMs to inform decision support. For health systems and policy-makers, missing data result in biased aggregate results, and thus bias group comparisons for evaluating interventions in real-world settings. Finally, missing data from PROMs threaten healthcare service buy-in as the cost of building, implementing, and maintaining PROM administration in clinical practice should be outweighed by the demonstrated value for patient care. Results from a study analyzed in 2019 revealed that while the clinical team engagement was associated with a 19.6% positive increase in PROMs completion rate, non-clinical engagement was associated with a 16.0% increase [8]. However, what influences patient adherence to PROM completion in clinical care is not clear from the evidence available.

In 2019, the International Society for Quality-of-Life Research (ISOQOL) Patient Engagement and QOL in Clinical Practice Special Interest Groups (SIGs) delivered a collaborative symposium and subsequent webinar in 2020 [16] to present and discuss experiences, knowledge and future directions for understanding and expanding patient engagement with routine use of PROMs to inform clinical care. Content during the original symposium included a presentation of a review of the literature and real-life case studies. A moderator led a discussion took place afterward. Each presenter has contributed a summary of their presentation, and to the synthesis of the overall recommendations informed by these presentations. An overview of the case studies is presented in Table 1.

**Table 1:** Characteristics of case studies

	Country	Study design	Health condition	PROM administration characteristics	Patient consent or usual care	PROM result usage
Case 1	Australia	Implementation pilot study using Integrated Promoting Action on Research Implementation in Health Services (iPARHIS) framework	Oncology	Online through touch screen computers in the waiting room	Patient consent	Results were made available to clinicians both at the time of consultation and on electronic health record for later use
Case 2	United Kingdom	Randomized control trial	Oncology	Weekly online reporting remotely	Patient consent	System provided patients with self-management advice immediately. Real time data of patient symptom were transferred to electronic health record for clinicians
Case 3	United States of America	Survey design	Orthopedic	Online patient portal/office-based tablet computer while in the waiting room	Usual care	Results were discussed with clinicians

## Results

### Case study 1—insights from an implementation study designed by patients, clinical teams, non-clinical teams, and researchers

#### Background

This was an implementation pilot study aiming to integrate a symptom PROM intervention in day-to-day patient care. Stakeholders, including patients, clinical teams, non-clinical teams, and researchers were engaged throughout all phases of the project.

#### Methods

This study was conducted in a medical oncology outpatients department of a major Australian quaternary referral hospital and tertiary teaching hospital. In this oncology department, a large number of specialist clinical teams manage services and care for complex patients traveling from around the state.

**Fig. 1:**
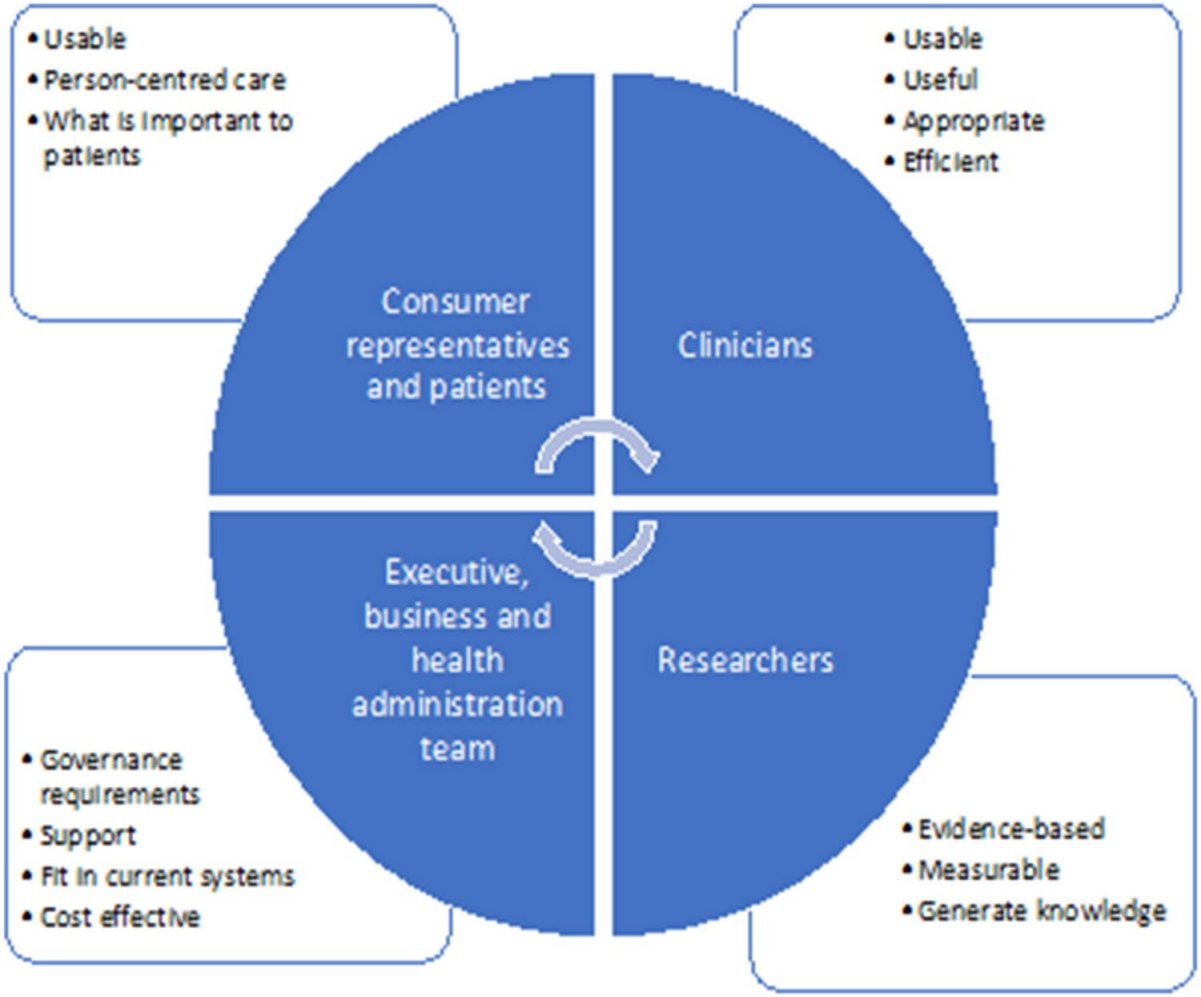
The use of co-design to address PROMs implementation requirements

Based on the integrated Promoting Action on Research Implementation in Health Services (iPARHIS) framework [17], participant engagement was initiated by asking all the key stakeholders (e.g., clinical teams, patients, consumer representatives, and other non-clinical teams) the question: What should the PROM look like for you? In Fig. 1, the key perspectives from each stakeholder are briefly presented. Using mixed methods (guided by iPARIHS constructs), data was collected pre-implementation (focus groups including 52 participants [18], process mapping, 12 stakeholder interviews, 83 clinical team surveys), during implementation (observational data, process mapping, PROM completion rates/response rates), and post-implementation for evaluation (72 clinical team surveys and focus groups including 30 participants). Plan Do Study Act cycles reported completion/response rates so that the co-design process could be used to further adapt the intervention so that it worked for them. The final design was a touchscreen computer in the oncology clinic waiting room for patients to complete the PROM to be used during clinical review and scanned later into the medical record [19]. Individual patient reports were made available to clinical teams both at the time of consultation and on the electronic health record for reference later. The intervention was piloted followed by an implementation evaluation phase [20].

#### Results

Patient engagement was measured by PROMs completion rates. Completion rates were dependent on events in the clinical setting, demonstrating that patient engagement is dynamic and contextual (70–100%). For example, when the touchscreen computer was relocated to accommodate a temporary change in the waiting room, completion rates dropped (< 50%), and when clinics were running behind schedule with longer waiting periods, completion rates were higher (90–100%). Additionally, data analyses showed that patients with high-grade persistent symptoms had a drop in completion rates over time when completing PROMs again on return visits. Qualitative responses from a patient when asked why they did not complete their PROMs included, “*What is the point? It’s just the same as last time”* and *“I can’t face it today.”*

The project also identified how expectations of the PROM changed over the course of the project implementation, between the initial design process, and when patients reflected back on its use. Early qualitative data captured patient opinions that they would support PROMs reporting because “*… [clinical teams] never seem to have time to ask [them] about [their] symptoms properly*.” During implementation, patients said that “*… if [they] did not have access to the touchscreen computer on arrival, [they would not bother to] seek it out and complete the [PROMs]*.” At the end of the pilot, patients reflected on the use of the PROM helped them explain things to the dietitian more easily, for example,* “I guess I just realized what to say from using it,” and “sometimes I don’t know if it is important or not.”*

#### Lessons learned

Adapting how the PROMs were presented and how patients were engaged is important for ensuring completion rates in the study clinics included in the study. We identified that each of the stakeholders brought an important perspective to the implementation of the PROM, and that this optimized the design. However, it would have been useful to  regularly evaluate patient expectations during “Plan Do Study Act” measurements, throughout implementation across different timepoints.

The relationship between patients and clinical teams impacted the PROM completion rates and the likelihood of discussing the PROM report. The completion of the PROM needs to be easy for patients, in a highly accessible location and the technology has to be suitable for the target group. In oncology, patients can be very sick and many have to manage their time and energy just to get through treatment. The effort of completing a PROM needs to be meaningful to their care and personal situation.

### Case study 2—longitudinal completion of PROMs: the eRAPID intervention during cancer treatment

#### Background

eRAPID is a PROMs centered eHealth intervention [21, 22] developed to monitor and support patients’ health throughout cancer treatment. The intervention was built on previous PROM interventions delivered in Leeds, UK [23, 24]. Embracing the increased use of mobile and online technology, the team worked closely with patients and oncology providers to explore how remote home-based monitoring could be used to enhance the routine and systematic assessment of treatment toxicity (such as fever, pain, neuropathy, fatigue).

Although a number of technology-based PROMs tools have been developed for the cancer setting, there is considerable variation in how they are designed and function [22]. The goal for eRAPID was to establish a practical system which met the needs of both patients and clinical teams. The system:

- Provides immediate severity tailored advice to patients on completion of the PROM reports (advice on how to self-manage and when to seek medical advice)
- Gives patients the option to view and monitor all of their personal symptom reports to show any changes over time
- Transfers symptom data real-time to electronic records for clinical teams to use

Overall acceptability of the system depends on the impact of technology and clinician willingness to include PROMs in their practice, in addition to patients’ motivation.

#### Methods

The main eRAPID trial was conducted [25] between 2015 and 2018 with patients commencing chemotherapy/systemic treatment for breast gynecological cancers. In the single-center 1:1 allocation randomized (at level of patient) controlled trial (RCT) (*N* = 508 participants) half the group received access to the eRAPID intervention in addition to usual care and were asked to complete the online symptom reports weekly for 18 weeks.

The trial was designed to evaluate if eRAPID provided additional benefit to usual care for patients’ quality of life (better symptom control) and clinical process outcomes (e.g., contact with the hospital, admissions, changes to treatment). In addition to exploring patient acceptability of the intervention through adherence to weekly online reporting, the trial also explored patient experience with measures of patient self-efficacy, by embedding interviews and end of study feedback surveys into the trial assessment.

#### Results

The results of the systemic RCT [26] contribute to the growing evidence highlighting the positive influence of PROMs in cancer care [1]. In terms of patient adherence to weekly symptom reports, around 70% of expected completions were achieved with a gradual reduction over the 18-week study period. From the patient feedback (interviews and written feedback), main reasons for not completing were forgetting, being too ill “*…sometimes I didn’t do it because I was too tired…’* or feeling well/not experiencing any issues, and finding the PROM completion/advice repetitive. Although there was a lot of positive feedback indicating patients found the system reassuring, and provided a connection with the hospital, many reported disappointment when data had not been used by clinical teams, ‘*Would have felt more relevant if someone had referred to the output at some stage or I knew the output was in my notes for the nurses or oncology team.’* For some this meant they stopped completing the assessments. We found PROMs completion was positively associated with clinical team use (based on frequency of clinical team feedback forms completed throughout the study period). The RCT study design meant some clinical teams saw limited numbers of intervention patients during the full trial. This meant some had little practice using the patient-reported measures in clinical assessments. It is important to note that some participants clearly found the process of completing and reporting symptoms personally useful, “*..gave me and my family more confidence to manage side effects especially early on in the treatment….”* and cathartic. Others explained their adherence to the online reporting was because they had made a commitment to the study and wanted to help future patients.

#### Lessons learned

Through the eRAPID trial we learnt that cancer patients were largely motivated and willing to complete remote online PROMs assessments during cancer treatment but had different motivations for doing so. Clinical team use of the outcome measures was an important to maintain patient engagement. Capturing qualitative insights on the use and value of the intervention was important for understanding patient experience and motivation to complete PROMs in instances where there is repeated PROM completion over time. Patients acknowledged the value of PROMs being embedded within a wider eHealth intervention (alongside self-management advice and ability to track symptoms) and this was a driver to complete PROMs for some. The RCT research context may have influenced how both patients and clinical teams engaged with the intervention and what was reported.

### Case study 3: a quality improvement study: evaluating the impact of collecting PROs on patient perceptions of orthopedic care

#### Background

In 2017, The University of Pittsburgh Medical Center (UPMC) expanded its collection and use of PROMs to include all general orthopedic care and subspecialties of sports medicine, hand and upper extremity, trauma, and musculoskeletal oncology.

With this expansion UPMC orthopedic clinical and non-clinical teams partnered on a quality improvement project to evaluate the impact that electronic collection and clinical use of PROMs information had on patient perceptions of their orthopedic care. The goal was to better understand the association between PROM collection and PROM clinical use with patient engagement and the patient experience.

UPMC had been collecting PROMs data on the academic side of the integrated system for years prior to the CMS total Joint Bundle in several clinical areas including primary care, rehabilitation, cancer and surgical specialties and due to this history, the administrative burden of the collection PROMs was reduced. Patients entered responses directly by completing questionnaires via an online patient portal or an office-based tablet computer, which are sent directly to their medical record while waiting for the clinical team to see them for their appointment.

#### Methods

The overall study findings have been reported elsewhere [27]. Patients aged 18 years and older who had an office visit between June 2017 and September 2017 with an email contact recorded were sent an invitation to complete an online survey about their visit.

The survey included the Altarum Consumer Engagement (ACE) questionnaire of 12 items across its three domains of commitment, informed choice, and navigation. Also included were two Clinician and Group Consumer Assessment of Healthcare Providers and Systems (CG-CAHPS) survey subsections consisting of 6 questions, which measured patient perceptions of physician communication and shared decision making and a final question asking if patients’ recalled completing the PROMs at the visit. Responses to the CG-CAHPS survey can be rated ‘top box’ (the most positive), ‘middle box’ (moderately positive) as ‘bottom box’ (least positive in domains [28]. A higher score meant that patients’ perceived better physician communication and shared decision making. Respondents were categorized into three groups: those that did not complete PROMs, those that completed PROMs but the doctor did not discuss them, and patients that completed PROMs and the doctor discussed them. CG-CAHPS provider communication questions were converted to a binary variable of top box or not top box. Group mean scores for ACE measure scores, percent top box doctor communication CAHPS scores, and percent positive shared decision making CAPHS scores were compared using user-defined formats to order output in an analysis of variance (PROC ANOVA) in Statistical Analysis System 9.4. Statistical significance was evaluated using the F test (*p* < 0.05) and groups were compared using Tukey’s procedure.

#### Results

Invitations were sent to 4,455 patients of which 558 people completed the survey (response rate 12.5%) of whom 150 (27%) reported not completing the PROM, 154 (28%) reported completing the PROMs but not discussing them with their doctor. 254 (46%) reported both completing the PROMs and discussing them with their doctor.

ACE commitment and ACE informed domain scores were not statistically different between groups of patients who completed PROMS and discussed their results with clinical team members or did not discuss results with them. ACE navigation scores were statistically significantly different (*p* = 0.045) and slightly higher (mean difference 1.02) in the group that reported completing PROMs and discussing them with their doctor compared to the group that reported completing PROMs but not discussed with their doctor. Which means that there was not a statistically different perception in commitment, informed choice and navigation.

Percent of CG-CAHPS ‘top box’ scores of physician communication were statistically different between groups (*p* < 0.0001). Patients who reported completing PROMs and discussing them with their doctor were 11.4% more likely to rate scores than those who reported not completing PROMs and 11.0% more likely to rate ‘top box’ scores than those who reported completing PROMs but not discussing them with their doctor. The group not completing PROMs and the group who completed PROMs but did not discuss them with their doctor were not statistically different. These results mean that those patients who discussed the PROMs with their doctor reported more positive scores for physician communication.

A similar pattern was seen in CG-CAHPS shared decision-making scores (*p* < 0.0001). Patients who reported completing PROMs and discussing them with their doctor were 16.5% more likely to report their doctors engaged in shared decision making than those who reported not completing PROMs and 15.9% more likely than those who reported completing PROMs but not discussing them with their doctor. The group not completing PROMs and the group who completed PROMs but did not discuss them with their doctor were not statistically different. These results mean that those patients who discussed the PROM with their doctor reported greater engagement in decision making.

#### Lessons learned

Patient perceptions of physician evaluation and discussion of PROM responses was significantly associated with patient ratings of better physician communication and shared decision-making. Patients who reported that their PROM responses were discussed during a visit were more likely to rate higher doctor communication and shared decision making, supporting evidence that PROMs support patient engagement.

## Discussion

**Box 1:** Summary of findings across case studies

Case Study 1:
• Involving clinical and non-clinical team members, researchers and patients in the design was advantageous
• Continuous evaluation of patient needs and expectations would have been beneficial
• Patient adherence appears to be influenced by the participation of clinical teams in the PROM findings
• The burden of PROM completion needs to be balanced with the functional impacts of illness
Case Study 2:
• Patients completed the PROM for a variety of reasons
• Clinical team engagement with PROM findings was a major influence to PROM completion by patients
• Self-management advice and symptom tracking were other motivators for PROM completion
• Completion rates for repeated measures dropped over time
Case Study 3:
• Patients reported greater physician communication and shared decision making when the PROM information was discussed
• PROM completion supports patient engagement in healthcare

Each of the case studies evaluated PROM completion with different lenses. Case Study 1 focused on an implementation science approach, Case Study 2 focusing on mechanisms underlying patient completion within a prospective trial, Case Study 3 focusing on the factors associated with PROM completion, and shared decision-making. The diversity of approaches exemplified in these case studies underscores the need to evaluate PROM integration taking into consideration the many influences to adherence, including healthcare systems, patients, clinical teams, and the chosen PROMs. Facilitators to PROM completion varied by study depending on which perspective was being assessed, ranging from how the PROMs were administered (Case Study 1), to engagement of clinical teams (Case Study 1, 2 and 3). The key findings across the case studies are presented in Box 1.

The purpose of the symposium presented at the 20th Annual ISOQOL conference was to describe the latest evidence, discuss common findings, and identify what questions remain. A key discussion point was the many complexities within the healthcare system (such as organizational readiness), patients (such as the capability to complete a measure), clinical teams (such as perceived relevance to care planning), and PROMs (such as technology) that directly influence patient adherence across diverse situations. There appears to be a lack of evidence about the many influences on patient adherence to PROMs completion, presenting opportunities to build a stronger evidence base. Case Study 1 was the only study that used a framework. Implementation science would be useful in future work to identify gaps and build structured guidance [29–31]. While equity was not discussed during the symposium, the literature has identified that this is important, and warrants more attention in future research [32].

Based on learnings from the literature as well as the case studies described here, we developed recommendations as key points for discussion and as research priorities moving forward. These recommendations are organized into findings relating to: (1) healthcare systems, (2) patients, (3) clinical teams, and (4) PROMs, and are presented in Table 2.

**Table 2:** Recommendations for future research

Healthcare systems: Approaches to PROM collection and corresponding clinical use have an overarching influence on how PROMs are introduced/collected, and ultimately how actively patients and clinical teams engage with them	1. Building on case study findings, researchers should identify implementation evidence that addresses patient adherence to completion of PROMs, including educational initiatives and health service outcomes 2. Conduct research on the design and features of electronic systems chosen for the healthcare system that influence patient adherence to PROMs [22]
Patients: Understanding the patient experience/expectations can help to inform appropriate education/support which will reinforce engagement	1. Work side by side with patient collaborators to co-design and co-evaluate PROM procedures in clinical care. This is an essential step to improve sustainable use of routine PROMs 2. Reflecting on patient quotes from Case Studies 1 and 2 about their energy levels and motivation for completing PROMs, it is important to include approaches that reduce patient burden. This can extend to the frequency of PROMs assessments, and the number of PROMs to be completed at one time. A brief and pragmatic PROM may improve adherence and accessibility
Clinical teams: The utility of PROMs and the tangible benefit or value from PROM completions is of paramount importance for clinical teams	1.Work toward identifying evidence-based ways to improve engagement with clinical teams, as the literature and case study findings indicate that this directly influences patient adherence to PROMs completion [33, 18, 34] 2. Further research is needed on training with teams to support patient adherence to PROMs completion) [35]. 3. From the literature, we identified that the number of patients seen per day by clinical teams can influence completion of PROMs in clinical care, with higher volume associated with lower completion rates [36, 20]. The underlying factors for why patient volume influences PROM completion are unknown, and future research should identify these factors and work to address the modifiable factors, including clinical team/time constraints, and treatment/wait times
PROMs: Much work relating to PROMs is collated through quality improvement projects and individual case studies across a range of healthcare systems	1. Studies define PROM completion rates differently (e.g., complete one time, complete throughout the entire study, complete some or all items). Therefore, we recommend improved standards of reporting in publications on patient PROM completion rates 2. Patients are more likely to complete PROMs provided at the beginning of a battery of questionnaires [12]. Work is needed to identify optimal thresholds for PROM length to reduce patient burden and ensure PROM completion, which could vary by patient health status. Consistent with the literature and Case Study 1, when time is made available from long waiting times in clinics, patient completion are improved, demonstrating that completion may depend largely on making the time available Identify evaluation measures that consider adherence to PROMs completion [37]

### Limitations

The work presented here is a description of the content and discussion from a symposium presented at the 20th ISOQOL conference. The content of this report intends to provide insights, rather than evidence.

## Conclusion

At the 20th Annual ISOQOL Conference, the clinical practice and patient engagement special interest groups aimed to share evidence and case studies about patient adherence to PROMs completion. Identified was that patient adherence to PROMs completion can be influenced by the healthcare system, clinical teams, patients, or by the PROMs being used. Structuring research questions through an implementation science lens may address these complexities.

## Data Availability

Availability of data and material on reasonable request to each case study team.

## Abbreviations

PRO: Patient-reported outcome
PROM: Patient-reported outcome measure
